# *Matrix Metalloproteinase-11* Gene Polymorphisms as a Risk for Hepatocellular Carcinoma Development in Egyptian Patients

**DOI:** 10.31557/APJCP.2020.21.12.3725

**Published:** 2020-12

**Authors:** Hind Saad, Magdy A-H Zahran, Olfat Hendy, Mohamed Abdel-Samiee, Hanan M Bedair, Eman Abdelsameea

**Affiliations:** 1 *Biochemistry Division, Department of Chemistry, Faculty of Science, Menoufia University, Shebein El-Kom, Egypt.*; 2 *Departement of Clinical Pathology, National Liver Institute, Menoufia University, Shebein El-Kom, Egypt. *; 3 *Chemistry Department, Faculty of Science, Menoufia University, Shebein El-Kom, Egypt. *; 4 *Departement of Hepatology and Gastroenterology, National Liver Institute, Menoufia University, Shebein El-Kom, Egypt. *

**Keywords:** Hepatocellular carcinoma, hepatitis C virus, single nucleotide polymorphism, MMP-11 polymorphisms

## Abstract

**Background:**

Chronic hepatitis C (CHC) virus infection is one of major risk factors of hepatocellular carcinoma (HCC) in Egypt, which is a major cause of cancer mortalityin the world. Matrix *metalloproteinase-11 (MMP-11)* has an important role in tumor migration and metastasis. Therefore, this study aimed to determine relation between *MMP-11* gene polymorphisms and risk of HCC development among Egyptian cirrhotic patients. Subjects and methods: Two hundred and sixty patients were included, 140 of them with HCC on top of CHC and 120 patients with post CHC liver cirrhosis (LC) as well as 140 subjects were enrolled in the study as healthy controls. Two single nucleotide polymorphisms (SNPs) rs738791 and rs738792 for *MMP-11* gene were done using real-time PCR.

**Results:**

Combination of CT and TT allele of rs738791 genotypes was more significantly frequent in HCC compared to LC patients and controls, however, a higher frequency of T allele was found in HCC patients compared to LC and controls. In spite of lake of significant difference between patient groups regarding the rs738792 genotypes, the CC genotype was considered a risk of developing portal vein thrombosis, and was associated with advanced tumor stage, increased tumor size, higher Cancer of the Liver Italian Program [CLIP] score, more advanced Barcelona stage [D] and with child Pugh class [C].

**Conclusion:**

Genetic variations in MMP-11 may be implicated in post HCV-HCC development and might be dependable biomarkers for HCC progression.

## Introduction

Hepatocellular carcinoma (HCC) is one of cancer causes of death all over the world (Gupta et al., 2019). HCC follows frequent diverse causes of damage to the liver such as chronic hepatitis C and B infection, chronic use of alcohol and nonalcoholic fatty liver disease (EASL guidelines, 2018). Chronic HCV infection represented 75 to 85% of infected persons. Nearly, 20% of them develop complications which include cirrhosis or HCC after 20 years of occurrence of infection (Josep et al., 2019).

Matrix metalloproteinases (MMPs) have an essential role in biological behaviors of most solid malignant tumors. MMPs include a family of endopeptidases which has the capacity of extracellular matrix proteins degradation and remodeling. They have important roles for progression of invasion and also metastasis of malignant solid tumors (Kessenbrock et al., 2010). MMP-11 is a member of MMPs family, also known astromelysin-3, is one of stromelysin subgroup that belongs to MMP superfamily (Zhang et al., 2016). It has been associated with HCC and its expression controls miR-125a-regulated proliferation, epithelial–mesenchymal transition and metastasis of HCC (Geervliet and Bansal, 2020). 

Genetic polymorphisms of MMP-11 have been found in different cancer types (Lin et al., 2015; Koleck et al., 2017). MMP-11 expression has been upregulated in human carcinomas as ovarian, breast, colorectal, lung and HCC (Rouyer et al., 1994; Bi et al., 2012) and minimal studies discussed its role in HCC.

Serum alfa fetoprotein (AFP) is the most common biomarker used in early HCC detection and prognostic factor for patient’s survival and/ or recurrence of tumor (Chaiteerakij et al., 2015). However, lake of its specificity and low sensitivity for HCC detection (Zhu et al., 2013), the search about new markers with more sensitivity and specificity is needed. Therefore the current case-control study was designed to determine the relation between MM-11 genotypes and risk of HCC development among Egyptian cirrhotic patients and to find its relation to HCC status. 

## Materials and Methods

All participants provided written informed consent prior starting in this study. The current study included: 260 patients were included, 140 of them with HCC on top of CHC and 120 patients with post CHC liver cirrhosis (LC). They were selected from the outpatient clinics of hepatology and gastroenterology department of the National Liver Institute (NLI), Menoufia University. HCC was diagnosed by the characteristic vascular enhancement pattern detected by multislice triphasic spiral CT scan and/ or MRI according to established diagnostic criteria (EASL guidelines, 2018). 

Clinical HCC classification was based on TNM and Barcelona- Clinic Liver Cancer (BCLC) staging systems. Diagnosis of cirrhosis was based on clinical findings, imaging studies (abdominal ultrasound) and laboratory results. Severity of cirrhosis was graded according to Child Pugh classification (Pugh et al., 1973). Additionally, 140 subjects with gender and age matched to patients were enrolled in the study as healthy controls.

Exclusion criteria: Patients with other causes of chronic liver disease, patients with other liver tumors (e.g. adenoma), HCC cases on top of co-infection with Hepatitis C and B, patients aged <18 years old, cases with chronic inflammatory diseases or autoimmune diseases were excluded from the study. Hematological malignancy and tumors of any organ other than the liver were of the exclusion criteria from the study.

The study was performed according to ethical standard of the responsible Institutional Committee and with the Helsinki Declaration of 1975 as revised in 2013. Either verbal or written consents were obtained from all subjects prior to enrollment in the study and the conventional data was collected from the patients’ files. The study protocol was in accordance with declaration of Helsinki and approval of the Scientific and Ethical Committee of NLI, Menoufia University.

*Laboratory investigations*

Ten ml of venous blood were drawn from all subjects included in this study by venipuncture from were divided into: 2ml was put in vacutainer tubes containing EDTA for molecular testing of polymorphism by real time PCR; 2ml was put in EDTA tube for complete blood count (CBC) and the 1.8ml was put in tube contains 0.2 ml of sodium citrate 3.8% for coagulation screening profile. The remaining volume was collected in plain vacutainer and allowed to clot and serum separated for liver function tests as alanine aminotransferase (ALT), aspartate aminotransferase (AST), albumin, total and direct bilirubin, hepatitis virus markers and AFP level.

CBC was done using Sysmex XT-1800i automated hematology analyzer (Sysmex, Japan), liver functions tests using Cobas e501 Auto analyzer (Roche, Germany), and AFP measurement using enzyme immunoassay based on electro-chemiluminescence immunoassay by Cobas e601 Auto analyzer (Roche). Prothrombin time (PT) and international normalized ratio (INR) value was done by BFT II fibrintimer. HBsAg and HCV antibodies were done by the ectroche-miluminescence immunoassay “ECLIA” using Cobas 6000 (e601 module).

*Genotyping of MMP-11*

Genomic DNA was extracted from EDTA samples using QIAamp DNA Mini Kit (catalog number, 51106, Qiagen, Santa Clarita, USA). MMP-11 was genotyped by real-time polymerase chain reaction (PCR) using fluorescent labeled probes. Single nucleotide polymorphism (SNP) was done for two MMP-11 variants (rs738791 and rs738792) as previously reported (Tecnologies, 2013). The ABI TaqMan allelic discrimination kit (catalog number 4351379, Applied Biosystems, Carlsbad, CA), briefly, the fluorescent labeled probes: GCCAGTGGTGGCCTGTGTCTACCAC[C/T] TCACCTCACTGAACCTGAGAGTCC (VIC dye for allele C, FAM for allele T) for rs738791. The probes: CATCCTCCTGCCTAGGACG[C/T]CCACCACCTCCATGCCGAGAGGAGG (VIC dye for allele T, FAM dye for allele C) was fluorescent probes for rs738792. The PCR fluorescence product was detected on a Rotor-Gene Q System (QIAGEN, GmbH- Germany) and the intensity of the fluorescence signal was analyzed by Rotor-Gene Q series software. The reaction was performed in a 96-well format in a total reaction volume of 25 µl using 20 ng of genomic DNA. The reaction was heated for 2 min at 50ºC, then 10 min at 95ºC, followed by 40 cycles of 95ºC for 15 sec and 60ºC for 1.5 min. The rs738791 and rs738792 genotyping results were recognized consequently by allele-separation dependent on relative enhancement of every particular fluorophore, adjusted for control Rox fluorophore.

*Statistical analysis*

Data were fed to the computer and analyzed using IBM SPSS software package version 22.0. (Armonk, NY: IBM Corp). Quantitative data were described mean, standard deviation (SD) or median and interquartile range (IQR). Chi-square test was used for categorical variables to compare between different groups. Mann Whitney test for abnormally distributed quantitative variables to compare between two studied groups. Kruskal Wallis test for abnormally distributed quantitative variables, to compare between more than two studied groups. Post Hoc for pairwise comparisons. Significance of the obtained results was judged at the level below 5%. Odd ratio (OR and 95% Confidence Interval of an event occurring in one risk group was calculated to the odds of it occurring in the non-risk group. The population of the studied sample was explored to find its equilibrium with Hardy-Weinberg equation by Hardy-Weinberg and regression to detect the most independent/ affecting factor for affecting HCC.

## Results

The gender and age were matched among the studied groups. DM was significantly more prevalent among patients with LC (p=0.032). There was a significant increase among studied groups as regards ALT, AST, total bilirubin and AFP compared to healthy control, while as regards serum albumin level and platelets among the studied groups, they were significantly decreased (p-value <0.001 for each). No significant difference was detected between HCC and LC groups regarding smoking (p = 0.407). Portal vein thrombosis is more frequent in HCC more than LC group (p<0.001); while no significant difference was found between HCC and liver cirrhosis patients regarding Child -Pugh score (p=0.073) (Table 1). 

No significant difference among studied groups was found regards the SNP rs738791 genotypes (p= 0.171). While the CT and TT genotypes combination was more frequent in HCC group compared to LC and control (p=0.049). Also, a higher frequency of T allele was detected in HCC patients (37.9%) compared to cirrhotic patients (34.2%) and healthy controls (28.6%) without statistical significant difference (Table 2).

No significant difference was observed among studied groups as regards SNP rs738792 genotype (TT, TC or CC), allele distributions (T or C) (p= 0.901 and 0.853 respectively) or combination of TC + CC genotypes (p=0.712) (Table 2).

The risk of hepatocellular carcinoma among different genotypes of rs 738791 (TT and CT genotype) was significantly increased by 2.077 fold (p1=0.018) and 1.731 fold (p1=0.032) respectively when compared to healthy control. Additionally, the patients carrying heterozygous (CT+TT) genotype and T allele had a significant increased 1.791 fold (p1=0.016) and 1.523 fold (p1= 0.020) respectively when compared to controls. While no significant differences were detected regarding rs738791 genotypes or allelic frequencies when comparing LC to HCC or healthy controls (Table 3). No statistically significant difference in the risk of HCC among all studied groups regarding genotypic or allelic frequencies of rs738792 polymorphism (Table 3).

The associations between MMP-11 genotypes and different studied parameters in HCC group revealed that, no statistically significant association between the CC, CT or TT of rs738791 genotype and all of age, sex, lymph node involvement, distant metastasis, TNM stage, AFP and tumor size. While, there was a significant association between different rs738791 genotypes and Barcelona stage (p< 0.001) as advanced Barcelona stages (C and D) was increased in presence of T allele in homozygous and heterozygous forms (CT+TT), CLIP score increased in cases with T allele in homozygous forms (TT) (p< 0.001). Also, the Child Pugh class B and C more increased in presence of T allele in homozygous and heterozygous forms (CT+TT) (p< 0.001) and PVT was more frequent in presence of T allele in homozygous and heterozygous forms (CT+TT) (p=0.008) (Table 4).

The HCC cases harboring TT, TC, and CC of rs738792 genotype showed no statistically significant association with each of sex, lymph node involvement, distant metastasis and AFP. While a significant difference was found between different genotypes and age (p< 0.001). The presence of C allele of rs738792 genotype in homozygous and heterozygous forms (CC+CT) had higher risk of developing PVT (p< 0.001), advanced tumor stage (p=0.002), increased focal lesion size >3 cm (p= 0.030), increased CLIP score (p<0.001), more advanced Barcelona stage [D] (p< 0.001) and more advanced liver failure with Child Pugh class [B and C] (p< 0.001) (Table 5). 

Table 6 showed that the observed distribution regarding genotype frequencies of rs738791 and rs738792 were consistent with the expected distribution in Hardy Weinberg equilibrium.

The univariate analysis conducted on potential risk factors of HCC, indicated that both AFP and T allele in homozygous and heterozygous forms (CT+TT) associated significantly (p<0.05) with the increased risk of HCC against controls (OR=1.940, 95% CI: 1.514 – 2.486, p<0.001; OR=1.792, 95% CI: 1.112 – 2.887, p=0.017). On multivariate analysis, all variables with p<0.05 were included, the AFP level was independent risk factor for HCC development (p<0.001) with a 1.946 fold while the CT/TT genotype appeared to be non-significantly associated with risk of HCC (OR=0.838, 95% CI: 0.379 – 1.852, p=0.662) (Table 7).

**Table 1:** Comparison of Demographic and Laboratory Data among the 3 Studied Groups

Parameters	HCC (n = 140)		LC (n = 120)		Controls (n = 140)		p- value
	No.	%	No.	%	No.	%	
Gender							
Male	107	76.4	78	65	103	73.6	0.108
Female	33	23.6	42	35	37	26.4	
	p_1_=0.054, p_2_=0.581, p_3_=0.134						
Age (years)							
Range	43.0 – 66.0		42.0 – 70.0		55.0 – 70.0		0.343
Mean ± SD	59.47 ± 4.72		58.85 ± 6.64		59.34 ± 3.58		
	p_1_=0.943, p_2_=0.108, p_3_=0.418						
DM							
No	84	60.00%	56	46.70%			0.032*
Yes	56	40.00%	64	53.30%			
Smoking							
No smokers	84	60.00%	78	65.00%			0.407
Smokers	56	40.00%	42	35.00%			
ALT (U/L)							
Mean ± SD	54.70 ± 44.45		38.20 ± 18.33		22.69 ± 4.06		<0.001*
	p_1_=0.013*, p_2_<0.001*, p_3_<0.001*						
AST (U/L)							
Mean ± SD	82.07 ± 78.24		63.33 ± 63.28		19.74 ± 4.73		<0.001*
	p_1_=0.321, p_2_<0.001*, p_3_<0.001*						
Albumin (g/dl)							
Mean ± SD	2.78 ± 0.88		2.58 ± 0.76		4.47 ± 0.22		<0.001*
	p_1_=0.166, p_2_<0.001*, p_3_<0.001*						
Total bilirubin (mg/dl)							
Median (IQR)	1.55 (1.10 – 5.80)		1.85 (0.92 – 4.02)		0.42(0.36 –0.60)		
	p_1_=0.430, p_2_<0.001*, p_3_<0.001*						
Platelets (×10^3^ /mm^3^)							
Mean ± SD	132.8 ± 90.47		127.7 ± 83.65		290.9 ± 67.41		<0.001*
	p_1_=0.767, p_2_<0.001*, p_3_<0.001*						
AFP (ng/dl)							<0.001*
Median (IQR)	152.25 (6.6 –1400)		2.56 (1.7 – 5.4)		2.10 (1.9 – 2.9)		
	p_1_<0.001*, p_2_<0.001*, p_3_=0.028*						
	N	%	N	%			
PVT			112	93.3			
No	82	58.6	8	6.7			<0.001*
Yes	58	41.4					
Child Pugh class							
A	48	34.3	26	21.7			
B	52	37.1	56	46.7			
C	40	28.6	38	31.7			

χ^2^, Chi square test; H, H for Kruskal Wallis test; MC, Monte Carlo; SD, Standard deviation; HCC, hepatocellular carcinoma; LC, liver cirrhosis; DM, Diabetes mellitus; ALT, alanine aminotransferase; AST, aspartate aminotransferase; AFP, Alpha fetoprotein; p_1_, value for comparing between HCC and liver cirrhosis; p_2_, value for comparing between HCC and control; p3, value for comparing between liver cirrhosis and control;*, Statistically significant at p <0.05

**Table 2:** Comparison of Genotypes and Allele Frequencies of MMP-11 (rs738791 and rs738792) among the 3 Studied Groups

Variable	HCC (n = 140)		LC (n = 120)		Controls (n = 140)		p –value
	No.	%	No.	%	No.	%	
rs738791 Genotype							
CC	52	37.1	50	41.7	72	51.4	0.171
CT	70	50	58	48.3	56	40	
TT	18	12.9	12	10.0	12	8.6	
CT+TT	88	62.9	70	58.3	68	48.6	0.049*
Allele frequency							
C	174	62.1	158	65.8	200	71.4	0.064
T	106	37.9	82	34.2	80	28.6	
rs738792 Genotype:							
TT	66	47.1	60	50.0	70	50	0.901
TC	62	44.3	48	40.0	60	42.9	
CC	12	8.6	12	10.0	10	7.1	
TC+CC	74	52.9	60	50.0	70	50	0.862
Allele frequency							
T	194	69.3	168	70.0	200	71.4	0.853
C	86	30.7	72	30.0	80	28.6	

χ^2^, Chi square test; SNP, Single nucleotide polymorphism; p, p value for comparing between the 3 studied groups;*, Statistically significant at p < 0.05.

**Table 3:** The Odds Ratios among the Three Studied Groups According to rs738791 and rs738792 Genotypes

Variable	p_1_	OR1 (CI. 95%)	p_2_	OR2 (CI. 95%)	p_3_	OR3 (CI. 95%)
rs738791 Genotype						
CC^®^	1			1		1
CT	0.032*	1.731 (1.049 − 2.856)	0.128	1.491 (0.891 − 2.496)	0.576	1.161(0.69 – 1.95)
TT	0.018	2.077 (0.921 − 4.682)	0.416	1.440 (0.599 − 3.464)	0.386	1.442(0.63 – 3.30)
CT+TT	0.016*	1.791 (1.11 – 2.89)	0.116	1.482 (0.91 – 2.42)	0.457	1.219 (0.73 – 1.99)
Allele frequency:						
C^®^	1					1
T	0.020*	1.523 (1.069 − 2.171)	0.17	1.298 (0.894 − 1.882)	0.383	1.738 (0.82 – 1.68)
rs738792 Genotype						
TT^®^	1			1		1
TC	0.713	1.096 (0.672 − 1.787)	0.792	0.933 (0.559 − 1.559)	0.54	1.174 (0.70 – 1.96)
CC	0.601	1.273 (0.515 − 3.143)	0.467	1.400 (0.565 − 3.469)	0.831	0.909 (0.38 – 2.18)
TC+CC	0.632	0.892 (0.56 – 1.43)	1	1.0 (0.61 – 1.62)	0.646	0.892(0.55 – 1.45)
Allele frequency:						
T^®^	1					1
C	0.579	1.108 (0.771 − 1.593)	0.721	1.071 (0.734 − 1.565)	0.86	1.034 (0.71 – 1.50)

CI, Confidence interval; OR1, Odds ratio for HCC and Control; P_1_, Comparison between HCC and Control; OR_2_, Odds ratio for LC and Control; P_2_, Comparison between LC and control; OR_3_, Odds ratio for HCC and LC; P_3_, Comparison between HCC and LC; p*, Statistically significant at p< 0.05

**Table 4:** Association between SNP rs738791 Genotype and Different Studied Parameters in HCC Group (n= 140)

	SNP rs738791 Genotype						p-value
	CC (n= 52)		CT (n= 70)		TT (n= 18)		
	No.	%	No.	%	No.	%	
Sex							
Male	41	78.8	52	74.3	14	77.8	0.833
Female	11	21.1	18	25.7	4	22.2	
Age (years)							
Range	43.0 – 65.0		50.0 – 66.0		55.0 – 65.0		0.747
Mean ± SD.	58.96 ± 5.28		59.60 ± 4.48		60.44 ± 3.94		
Lymph nodes							
No	44	84.6	58	82.9	18	100	0.172
Yes	8	15.4	12	17.1	0	0	
Metastasis							
No	52	100	66	94.3	18	100	MCp=
Yes	0	0	4	5.7	0	0	0.184
T (Tumor stage)							
T1	22	42.3	22	31.4	4	22.2	MCp=
T2	24	46.2	26	37.1	6	33.3	0.056
T3	6	11.5	20	28.6	8	44.4	
T4	0	0	2	2.9	0	0	
PVT							
No	38	73.1	38	54.3	6	33.3	0.008*
Yes	14	26.9	32	45.7	12	66.7	
AFP (ng/ml)							
Median	41.79		287		405.2		0.097
Focal lesion size							0.073
<3 cm	22	42.3	14	20	4	22.2	
>3 cm	30	57.7	56	80	14	77.8	
CLIP score for HCC							
0	10	19.2	2	2.9	0	0	MCp
1	14	26.9	10	14.3	0	0	<0.001*
2	10	19.2	20	28.6	2	11.1	
3	8	15.4	14	20	4	22.2	
4	8	15.4	12	17.1	4	22.2	
5	2	3.8	6	8.6	8	44.4	
6	0	0	6	8.6	0	0	
BCLC score for HCC							
A	14	26.9	10	14.3	0	0	<0.001*
B	22	42.3	16	22.9	2	11.1	
C	10	19.2	20	28.6	6	33.3	
D	6	11.5	24	34.3	10	55.6	
Child score							
A	24	46.2	24	34.3	0	0	<0.001*
B	22	42.3	22	31.4	8	44.4	
C	6	11.5	24	34.3	10	55.6	

χ^2^, Chi square test MC: Monte Carlo; H, H for Kruskal Wallis test; SNP, single nucleotide polymorphism; PVT, portal vein thrombosis; CLIP score, Cancer of the Liver Italian Program Score; BCLC stage, Barcelona clinic liver cancer stage; TNM, Tumor/node/ metastasis; *, Statistically significant at p < 0.05

**Table 5:** Association between SNP rs738792 Genotype and Different Studied Parameters in HCC Group (n= 140)

Parametres	SNP rs738792 Genotype						P- value
	TT (n= 66)		TC (n= 62)		CC (n= 12)		
	No.	%	No.	%	No.	%	
Sex							
Male	52	78.8	45	72.6	10	83.3	0.597
Female	14	21.2	17	27.4	2	16.7	
Age (years)							
Range	51.0 – 66.0		50.0 – 65.0		43.0 – 60.0		<0.001*
Mean ± SD.	60.42 ± 3.91		59.87 ± 4.13		52.17 ± 5.70		
Lymph nodes							
No	56	84.8	52	83.9	12	100	0.331
Yes	10	15.2	10	16.1	0	0	
Metastasis							
No	62	93.9	62	100	12	100	
Yes	4	6.1	0	0	0	0	MCp=0.186
T (Tumor stage)							
T1	32	48.5	16	25.8	0	0	
T2	20	30.3	28	45.2	8	66.7	
T3	12	18.2	18	29	4	33.3	MCp=0.002*
T4	2	3	0	0	0	0	
PVT							
No	50	75.8	32	51.6	0	0	<0.001*
Yes	16	24.2	30	48.4	12	100	
AFP (ng/ml)							0.584
Median	101.9		245		914.9		
Focal lesion size							
<3 cm	24	36.4	16	25.8	0	0	0.030*
>3 cm	42	63.6	46	74.2	12	100	
CLIP score for HCC							
0	12	18.2	0	0	0	0	
1	20	30.3	4	6.5	0	0	MCp
2	18	27.3	14	22.6	0	0	
3	12	18.2	14	22.6	0	0	<0.001*
4	4	6.1	16	25.8	4	33.3	
5	0	0	10	16.1	6	50	
6	0	0	4	6.5	2	16.7	
BCLC score for HCC							
A	22	33.3	2	3.2	0	0	<0.001*
B	24	36.4	16	25.8	0	0	
C	18	27.3	18	29	0	0	
D	2	3	26	41.9	12	100	
Child score							
A	48	72.7	0	0	0	0	MCp
B	16	24.2	36	58.1	0	0	<0.001*
C	2	3	26	41.9	12	100	

χ^2^, Chi square test; MC, Monte Carlo; H, H for Kruskal Wallis test; PVT, portal vein thrombosis; BCLC stage, Barcelona clinic liver cancer stage; TNM, Tumor/node/ metastasis; *, Statistically significant at p <0.05.

**Table 6:** Distribution of Observed SNP rs738791 and rs738792 Genotypes Frequencies and Their Consistent with Hardy-Weinberg

Variable	Observed	Expected	χ^2^ Test	p value
SNP rs738791 Genotype				
HCC (n= 140)				
CC	52	54.1	0.55	0.458
CT	70	65.9		
TT	18	20.1		
Cirrhosis (n= 120)				
CC	50	52	0.664	0.415
CT	58	54		
TT	12	14		
Controls (n= 140)				
CC	72	71.4	0.056	0.812
CT	56	57.1		
TT	12	11.4		
Total (n= 400)				
CC	174	176.9	0.421	0.516
CT	184	178.2		
TT	42	44.9		
SNP rs738792 Genotype				
HCC (n= 140)				
TT	66	67.2	0.23	0.631
TC	62	59.6		
CC	12	13.2		
Cirrhosis (n= 120)				
TT	60	58.8	0.272	0.601
TC	48	50.4		
CC	12	10.8		
Controls (n= 140)				
TT	70	71.4	0.35	0.554
TC	60	57.1		
CC	10	11.4		
Total (n= 400)				
TT	196	197.4	0.112	0.737
TC	170	167.2		
CC	34	35.4		

If p< 0.05 it is not consistent with Hardy-Weinberg Equilibrium (HWE)

**Table 7:** Univariate and Multivariate Analysis for All Studied Parameters Affecting HCC Group Versus Control

Variable	Univariate		#Multivariate	
	p	OR (95%C.I)	P	OR (95%C.I)
Sex (male)	0.581	0.859 (0.499 – 1.476)		
Age (years)	0.797	1.007 (0.952 – 1.066)		
Smoking	0.06	1.610 (0.979 – 2.646)		
AFP	<0.001*	1.940 (1.514 – 2.486)	<0.001*	1.946 (1.516 – 2.499)
SNP rs738791 Genotype (CT+TT)	0.017*	1.792 (1.112 – 2.887)	0.662	0.838 (0.379 – 1.852)

## Discussion

Cirrhosis is most commonly caused by chronic hepatitis C infection and it is one of the major risk factors of HCC development (Berry et al., 2019). Matrix metalloproteinase-11 (MMP-11) has an important role in tumor migration and metastasis. Therefore, this study was designed to evaluate the value of two single nucleotide polymorphisms (SNPs) rs738791 and rs738792 for MMP-11 as a risk factor of HCC development among Egyptian cirrhotic patients. Two hundred sixty patients were included in the study, 140 of them with HCC on top of CHC and 120 patients with post CHC liver cirrhosis (LC) as well as 140 subjects were enrolled in the study as healthy controls. 

Diabetes mellitus (DM) in our study was significantly more prevalent among patients with LC than in those with HCC. The current data were in contrary with a study carried out by Hassan et al. (2010) who reported that diabetes appears to increase risk of HCC and this risk is correlated with a long duration of having diabetes.

In the current study, platelet count and albumin are significantly lower in both cirrhotic and HCC patients compared to control group. This was in agreement with Franca et al., (2004) who reported different theories about thrombocytopenia in chronic liver diseases. They include sequestration of platelets in spleen due to portal hypertension, decreased thrombopoietin levels, bone marrow suppression because of underlying liver disease present and auto-antibody destruction of platelets. 

Dufour et al., (2000) and Harris, (2005) stated that serum level of albumin is an excellent marker of synthetic function of the liver in patients with chronic liver disease which indicates poor liver function. The most common cause for a low albumin is chronic liver failure due to presence of cirrhosis. 

In the present study, a significant increase in aminotransferases (ALT and AST) was detected in HCC group more than that in cirrhosis group compared to the control group. This result was compatible with the previously stated that liver tests were elevated significantly in patients with HCC in comparison to chronic liver disease (Zekri et al., 2010). Also, in the present study, there was no significant difference of these parameters between cirrhosis and HCC groups. This finding was completely in agreement with the previously reported that hepatic functions cannot distinguish between HCC and cirrhosis (Silva et al., 2008). AST and ALT measure the intracellular hepatic enzymes concentration that have released into circulation and act as hepatocyte injury marker. Alkaline phosphatase, bilirubin and gamma glutamyl transpeptidase serve as markers of cholestasis and biliary function (Harris, 2005).

In our study, we found that patients with HCC had the highest level of AFP compared to cirrhotic patients and controls with a range up to 1400 ng/ml. This was in agreement with a study done by Stewart in 2008 who found that AFP is increased in HCC patients and > 90% of such patients have elevated levels in the range of 400 -500 ng/ml (Stewart, 2008). Also AFP level was significantly higher in cirrhotic patients in comparison with controls in our study, which agreed with Wang et al., (2005) and Tai et al., (2009) who reported in their study raised levels of AFP in cirrhotic patients without HCC. Di Bisceglie et al., (2005) observed that AFP production is triggered by presence of inflammation, occurrence of hepatocellular injury and necrosis, possibly as a result of increased turnover of hepatocyte. 

Also, Mousa et al., (2012) reported that elevated levels of serum AFP may be a result of altered hepatocyte-hepatocyte interaction which is associated with a loss of normally present architectural arrangements rather than active regeneration or necrosis. Chaiteerakij et al., (2015) reported that, serum level of AFP is considered as early diagnostic and prognostic biomarker for HCC, and it is an independent risk predictor of pathological grade, disease progression, patient survival and tumor recurrence (Bai et al., 2017).

Moreover the MMP-1 polymorphism in the present study, no significant difference among studied groups was found as regards the SNP rs738791 genotypes. While the CT and TT genotypes combination was more frequent in HCC group compared to LC and control. Also, a higher frequency of T allele was detected in HCC patients (37.9%) compared to cirrhotic patients (34.2%) and healthy controls (28.6%) without statistical significant difference. No significant difference was observed among studied groups as regards SNP rs738792 genotype (TT, TC or CC), allele distributions (T or C) or combination of TC + CC genotypes.

The risk of hepatocellular carcinoma among different genotypes of rs 738791 (TT and CT genotype) was significantly increased by 2.077 fold and 1.731 fold respectively when compared to healthy control. Additionally, the patients carrying heterozygous (CT+TT) genotype and T allele had a significant increased 1.791 fold and 1.523 fold respectively when compared to controls. While no significant differences were detected regarding rs738791 genotypes or allelic frequencies when comparing LC to HCC or healthy controls. These findings suggest that different MMP-11 polymorphisms may play different roles in development of cancer.

In contrast, no statistically significant difference among all studied groups regarding genotypic or allelic frequencies of rs738792 polymorphism was found. These results were compatible with the previously reported study Wang et al., (2018) as they found that there was no significant difference as regards HCC patients with the rs738792 polymorphisms when compared with healthy controls. 

The associations between MMP-11 genotypes and different studied parameters in HCC group revealed that, no statistically significant association between the CC, CT or TT of rs738791 genotype and all of age, sex, lymph node involvement, distant metastasis, TNM stage, AFP and tumor size. These results were also obtained by Wang et al., (2018) who revealed that no significant associations between AFP serum level in HCC patients and genotypes of any MMP-11 SNPs.

There was a significant association between different rs738791 genotypes and Barcelona stage as advanced Barcelona stages (C and D) was increased in presence of T allele in homozygous and heterozygous forms (CT+TT), CLIP score increased in cases with T allele in homozygous forms (TT). Also, the Child Pugh class B and C more increased in presence of T allele in homozygous and heterozygous forms (CT+TT) and PVT was more frequent in presence of T allele in homozygous and heterozygous forms (CT+TT). MMP-1 high expression proved to be a risk factor for tumor recurrence and independent molecular marker of prognosis in HCC and may become a novel target in the strategies for the prediction of tumor progression and prognosis of this disease (Yassen et al., 2013). 

On the other hand, the HCC cases harboring TT, TC, and CC of rs738792 genotype showed no statistically significant association with each of sex, lymph node involvement, distant metastasis and AFP. While significant difference was found between different genotypes and age. The presence of C allele of rs738792 genotype in homozygous and heterozygous forms (CC+CT) had higher risk of developing PVT, advanced tumor stage, increased focal lesion size >3 cm, increased CLIP score, more advanced Barcelona stage [D] and more advanced liver failure with Child Pugh class [B and C].These results agreed with Wang et al. (2018) who reported that no significant associations between the levels of HCC clinical pathologic markers and genotypes of any MMP-11 SNPs.

The current study revealed that the observed distribution regarding genotype frequencies of rs738791 and rs738792 and Hardy-Weinberg, the distribution was consistent with the expected distribution in Hardy Weinberg equilibrium.

The univariate analysis conducted on potential risk factors of HCC in our work indicated that both AFP and T allele of SNP rs 738791 in homozygous and heterozygous forms (CT+TT) was associated significantly (p<0.05) with the increased risk of HCC against controls (OR=1.940, 95% CI: 1.514 – 2.486, p<0.001; OR=1.792, 95% CI: 1.112 – 2.887, p<0.017). Also, Wang et al., 2018 reported that after potential confounders adjustment, subjects with combined CT and TT of MMP-11 rs738791 polymorphism had a 1.389-fold- ( %95 CI: 1.004-1.921; p < 0.05) higher risk of HCC development compared to those with homozygotes C/C.

In conclusion, we found that the genetic polymorphisms in the *Matrix metalloproteinases-11 (MMP-11) *gene: rs738791, but not rs738792 was significantly associated with risk of HCC development in Egyptian patients with chronic HCV infection. Carriers of the CT+TT allele of the rs738791 variant were at greater risk of HCC compared with wild-type (CC) carriers. Furthermore, the presence of C allele of rs738792 genotype in homozygous and heterozygous forms (CC+CT) had higher risk of developing PVT, increased tumor size, advanced tumor stages, higher CLIP score, advanced Barcelona stage and more advanced liver cell failure. Future large scale studies are recommended to clarify the role of other genetic variation in the *MMP-11* gene that may help in understanding the pathogenesis of HCC and to be targeting for therapy.
